# Probing ultrafast ππ*/nπ* internal conversion in organic chromophores via K-edge resonant absorption

**DOI:** 10.1038/s41467-017-00069-7

**Published:** 2017-06-22

**Authors:** T. J. A. Wolf, R. H. Myhre, J. P. Cryan, S. Coriani, R. J. Squibb, A. Battistoni, N. Berrah, C. Bostedt, P. Bucksbaum, G. Coslovich, R. Feifel, K. J. Gaffney, J. Grilj, T. J. Martinez, S. Miyabe, S. P. Moeller, M. Mucke, A. Natan, R. Obaid, T. Osipov, O. Plekan, S. Wang, H. Koch, M. Gühr

**Affiliations:** 1 Stanford PULSE Institute, SLAC National Accelerator Laboratory, 2575 Sand Hill Road, Menlo Park, CA 94025 USA; 2 Department of Chemistry, Norwegian University of Science and Technology, NO-7491 Trondheim, Norway; 3 Dipartimento di Scienze Chimiche e Farmaceutiche, Università degli Studi di Trieste, Piazzale Europa 1, Trieste, IT-34127 Italy; 4 Department of Chemistry, Technical University of Denmark, 2800 Kongens Lyngby, Denmark; 5 Department of Physics, University of Gothenburg, SE-412 96 Gothenburg, Sweden; 6 Department of Physics, University of Connecticut, 2152 Hillside Road, Storrs, CT, 06269 USA; 7 Linac Coherent Light Source, SLAC National Accelerator Laboratory, 2575 Sand Hill Road, Menlo Park, CA 94025 USA; 8 Argonne National Laboratory, 9700 Cass Avenue, Lemont, IL 60439 USA; 9 Department of Physics and Astronomy, Northwestern University, 2145 Sheridan Road, Evanston, IL 60208 USA; 10 Department of Physics, Stanford University, 382 Via Pueblo Mall, Stanford, CA 94305 USA; 11 Stanford Synchrotron Radiation Lightsource, SLAC National Accelerator Laboratory, 2575 Sand Hill Road, Menlo Park, CA 94025 USA; 12 Laboratory of Ultrafast Spectroscopy, Ecole Polytechnique Federal de Lausanne, Lausanne, CH-1015 Switzerland; 13 Department of Chemistry, Stanford University, 333 Campus Drive, Stanford, CA 94305 USA; 14 Laser Technology Laboratory, RIKEN, Wako, Saitama 351-0198 Japan; 15 Department of Physics and Astronomy, Uppsala University, Box 516, SE-751 20 Uppsala, Sweden; 16 Elettra-Sincrotrone Trieste, Strada Statale 14-km 163,5 AREA Science Park, IT-34149 Basovizza Trieste, Italy; 17 Institut für Physik und Astronomie, Universität Potsdam, Karl-Liebknecht-Straße 24/25, DE-14476 Potsdam, Germany

## Abstract

Many photoinduced processes including photosynthesis and human vision happen in organic molecules and involve coupled femtosecond dynamics of nuclei and electrons. Organic molecules with heteroatoms often possess an important excited-state relaxation channel from an optically allowed ππ* to a dark nπ* state. The ππ*/nπ* internal conversion is difficult to investigate, as most spectroscopic methods are not exclusively sensitive to changes in the excited-state electronic structure. Here, we report achieving the required sensitivity by exploiting the element and site specificity of near-edge soft X-ray absorption spectroscopy. As a hole forms in the *n* orbital during ππ*/nπ* internal conversion, the absorption spectrum at the heteroatom K-edge exhibits an additional resonance. We demonstrate the concept using the nucleobase thymine at the oxygen K-edge, and unambiguously show that ππ*/nπ* internal conversion takes place within (60 ± 30) fs. High-level-coupled cluster calculations confirm the method’s impressive electronic structure sensitivity for excited-state investigations.

## Abstract (web summary)

Many photo-induced processes such as photosynthesis occur in organic molecules, but their femtosecond excited-state dynamics are difficult to track. Here, the authors exploit the element and site selectivity of soft X-ray absorption to sensitively follow the ultrafast ππ*/nπ* electronic relaxation of hetero-organic molecules.

## Introduction

The efficient conversion of light into other forms of energy has a key role in many processes such as photosynthesis or human vision^1, 2^. It is well established that the efficiency of these processes is facilitated by coupled ultrafast electronic and nuclear dynamics that cannot be described using the Born–Oppenheimer approximation (BOA). The breakdown of the BOA makes the fundamental details of such mechanisms notoriously difficult to understand: they proceed on ultrafast timescales and occur mostly at positions where potential energy surfaces come close or even intersect. From an experimental point of view, it is highly desirable to access the nuclear geometry and the electronic degrees of freedom separately, to compare them to quantum simulations. The transient nuclear geometry can be best studied by time-resolved diffraction techniques using short X-ray^3, 4^ or electron pulses^5^.

X-ray absorption spectroscopy has been known for a long time to be an element and site-specific probe for local electronic structure of organic molecules and the charge states and local environment of transition metal sites^6^. The element and site specificity originates from excitation of inner electrons, which are strongly confined around the specific nucleus, into empty valence molecular orbitals. As the core electron energy levels often lie 10*s* and 100*s* of eVs apart, a specific site in a molecule can be probed by selection of the incident photon energy. Fueled by the development of short X-ray pulse sources^7^ and the growing interest in transition metal-based photocatalysts and photosensitizers, transient X-ray spectroscopy was very successfully applied to the K- and L-edges of the active transition metal centers in those compounds^8–11^. These studies showed an impressive sensitivity to transiently populated electronic states involving the metal center, e.g., metal-to-ligand charge transfer (MLCT) states.

Internal conversion between excited states of different electronic character is also a crucial path for photoenergy conversion in organic molecules. Organic chromophores exhibit strongly absorbing ππ* excited states, which can be described in a single electron Hartree–Fock (HF) picture as an electron–hole pair in a formerly occupied and an unoccupied molecular orbital (MO), both with *π* symmetry. Many of these chromophores, like azo-switches^12, 13^, nucleobases^14–16^, and amino acids^17^, also contain heteroatoms with electron lone pairs. They therefore exhibit nπ* excited states, with a hole in a heteroatom-centered lone pair (*n*) orbital and an electron in a π* orbital. Unlike ππ* excited states, nπ* states are usually not directly accessible because of low absorption cross-sections from the ground state. It is therefore crucial to directly monitor the ππ*/nπ* internal conversion as it provides essential photochemical pathways for reactions like *cis*–*trans* isomerization, and intersystem crossing to the triplet manifold of electronic states governed by the El Sayed selection rules^18^.

**Fig. 1:**
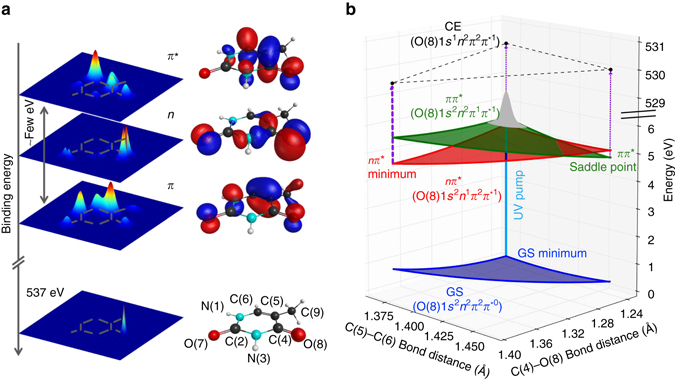
Excited states of thymine and their electronic characters. **a** Isosurface representations (*right*) and electron density projections onto the molecular plane for the three valence orbitals (Hartree–Fock/6-311 G) involved in the characters of the two lowest lying excited states of thymine and a core orbital localized at oxygen(8). The electron density at the position of the core orbital differs strongly for the different valence orbitals. **b** Results from our coupled cluster investigation of the excited-state topology along the two most relevant degrees of freedom for relaxation into the nπ* minimum. All states are labeled with their electron configuration. Ultraviolet (UV) excitation of the ground state (GS) places a nuclear wavepacket (*gray*) on the ππ* excited state. It relaxes through a conical intersection to a minimum in the nπ* excited state. According to our calculations, only one core-excited state (CE) characterized by an excitation at O(8) is relevant for interpretation of our experimental results

The preferential localization of the n orbital at the heteroatom has wide-reaching implications for resonant core level spectroscopy. In general, near edge X-ray absorption fine structure (NEXAFS) spectra show isolated features from resonant states below the core ionization edge of an element. Those features are because of transitions from this element’s core orbital to unoccupied valence orbitals, e.g., a π* orbital. The core-to-valence absorption cross-section is strongly dependent on the spatial overlap between the confined core and the empty valence orbital^6, 19, 20^ (Fig. 1a). In the case of excited states, the electron hole in a formerly occupied valence orbital enables an additional NEXAFS resonance. The spatial overlap makes a 1*s*–*n* transition from the strongly localized heteroatom 1s core level to the electron hole of an nπ* state more intense than the 1*s*–*π* transition to the delocalized *π* hole of a ππ* state. The same effect is observable in the strong sensitivity of time-resolved (TR) NEXAFS spectroscopy to MLCT states in transition metal complexes. The TR-NEXAFS signature from a photoexcited ππ* state is therefore expected to be weak and to transform into a strong nπ*-state signature as the molecule undergoes ππ*/nπ* internal conversion, largely independent of geometry changes during the dynamics.

One benchmark molecule for the sensitivity of TR-NEXAFS spectroscopy to the ππ*/nπ* transition is the chromophore thymine, as there is a rich literature on its excited states (see^14, 15, 21^ and citations therein). Thymine exhibits two high-lying occupied MOs, an oxygen-localized *n*-orbital and a delocalized *π*-orbital (Fig. 1a). Its lowest unoccupied MO (π*) is similar to the *π* MO in delocalization. The molecule can be excited at 267 nm to a ππ* state; the lower-lying nπ* state is optically dark.

The ππ* relaxation in thymine has been experimentally investigated using many methods available in ultrafast technology^14, 15, 21^ including our own study, where we investigated excited-state nonresonant Auger spectra at the oxygen K-edge^22^. It is challenging to attribute the experimental signatures of sub-100 fs thymine dynamics from ultrafast photoelectron^16^, photoion^23^, absorption, or Auger electron spectroscopy^22^ unambiguously to a particular process, like internal conversion, as changes in both the electronic structure and the nuclear geometry influence the observables. Interpretations of past experimental data involving valence electrons relied heavily on simulations. However, theoretical investigations do not agree on a common prediction of the excited-state dynamics^14^. Several wavepacket simulations predict nuclear relaxation into a local minimum of the ππ* state within 100 fs followed by slower ππ*/nπ*^24, 25^ or even ππ*/ground state^26^ internal conversion over a barrier. Others predict barrierless, sub-100 fs ππ*/nπ* relaxation^27^ or direct ππ*/ground-state relaxation within few hundred fs^28^. It is therefore unclear, if and on which timescale the nπ* state is accessed during the relaxation dynamics.

In this work, we demonstrate application of the well-established state sensitivity of transition metal X-ray absorption spectroscopy to time resolved spectroscopy of electronic relaxation in organic molecules, closing an important gap in the photochemistry that can be investigated. We demonstrate at the example of thymine that our TR-NEXAFS technique is strongly and selectively sensitive to the ultrafast ππ*/nπ* internal conversion. We prove the existence of the relaxation channel into the nπ* state, its ultrafast population and depopulation. Employing computationally demanding, but quantitative high-level-coupled cluster (CC) simulations^29–31^, which are still unavailable for transition metal complexes, we confirm our spectroscopic attribution based on orbital localization.

## Results

### Ground-state and excited-state NEXAFS spectra

**Fig. 2:**
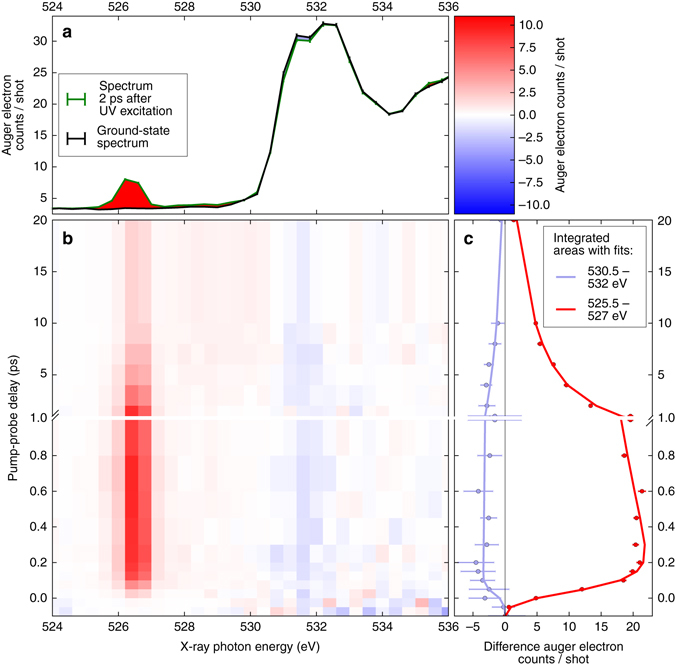
Transient oxygen-edge absorption spectra of thymine. **a** Representative near-edge absorption fine structure (NEXAFS) spectra 2 ps after ultraviolet (UV) excitation and without UV excitation. UV-induced increase in intensity is marked *red*, UV-induced decrease is *light blue*. UV excitation leads to the appearance of a new spectral feature around 526.4 eV and a bleach of the ground state π* resonance at 531.4 eV. **b** False-color plot of time-dependent NEXAFS difference spectra (see color bar in the *upper right* corner). The UV-induced features at 526.4 and 531.4 eV are clearly visible throughout the positive pump-probe delays. **c** Time-dependence of the UV-induced features with fits based on a rate equation model. All error bars represent the standard error of mean

The experimental ground-state NEXAFS spectrum of thymine is shown in black in Fig. 2a. It exhibits a double peak π* resonance. On the basis of our CC calculations, and in agreement with earlier studies^32^, we assign the lower energy peak at 531.4 eV to a linear combination of HF single electron excitations from the O(8) 1*s* orbital to several unoccupied π* orbitals with significant contributions from the aforementioned π* MO. The linear combination is such that the core excited state possesses a high degree of electron localization at O(8), thus the strong absorption cross-section in the Mbarn regime (see a discussion in Supplementary Note 1 and Supplementary Fig. 1). The higher energy peak at 532.2 eV corresponds to an excitation from the O(7) 1*s* orbital to a different linear combination of π* MOs^32^. The increase in intensity at photon energies beyond the π* resonances is predominantly because of a smooth feature of K-edge ionization at 537 eV and additional weak resonant transitions^32^.

The NEXAFS spectrum taken 2 ps after ultraviolet (UV) excitation is shown in green in Fig. 2a. It is a superposition of the excited-state spectrum and the ground-state spectrum, which is weakened by transfer of an estimated 13% of the population (Supplementary Notes 2–4) to the excited state. The excited-state spectrum is redshifted to lower photon energies with respect to the ground state, which is obvious by the background-free signature at 526.4 eV. This signature must be a new core excitation channel either to the *n* or *π* electron hole. An additional signature of UV excitation, the intensity reduction in the area of the π* resonances, is the result of a bleach of the ground-state spectrum almost entirely compensated by the redshift of the smooth K-edge ionization feature in the excited-state spectrum. The effect is therefore only visible, where the ground state exhibits the strongest intensity modulations, i.e., the π* resonance. It is therefore a direct signature of the ground-state depopulation and largely independent of any following excited-state dynamics.

### Signature of ultrafast ππ*/nπ* internal conversion

**Fig. 3:**
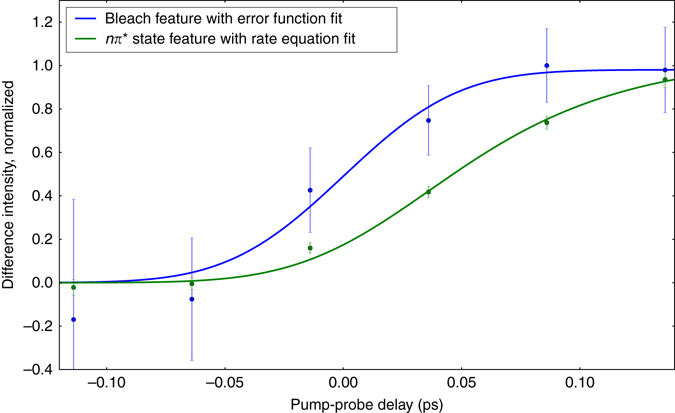
Delay between the onsets of ground-state bleach and excited-state signature. The experimental data for the bleach signal (*blue*) are extracted from a region of interest in the Auger spectra from the photon energy region, where the bleach is observable in the near-edge absorption fine structure spectra. For better comparison with the corresponding data set from the region of the nπ* feature (*green*), the bleach data set is inverted. Both data sets are normalized to the maximum modulation. The time-dependence of the nπ* feature is fitted with the rate equation model. The delay between the bleach and the nπ* feature is clearly visible. All error bars represent the standard error of mean

The time-dependence of the difference signal (X-ray absorption with UV minus X-ray absorption without UV) is shown in Fig. 2b. The spectrally integrated time trends of the ground-state bleach and excited-state feature are shown in Fig. 2c. The temporal onset of the excited-state feature exhibits a delay ((60 ± 30) fs according to a rate equation fit, see Supplementary Note 2) with respect to the temporal overlap between UV and X-ray pulses, which is marked by the bleach onset. To confirm the fit of the rather noisy bleach feature, we defined a narrower integration region in the Auger spectra based on best signal to noise for the bleach feature. The resulting curves and fits are shown in Fig. 3. The intensity of the 526.4 eV feature is only because of 13% of the population in the ground-state NEXAFS spectrum. Its absorption cross-section is, thus, similar to the π* resonance. Therefore, it must be likewise due to a localized transition, which is the signature of the nπ* state, not the ππ* state. Accordingly, the delay of the nπ* signature of (60 ± 30) fs directly reflects the nuclear wavepacket dynamics to access the ππ*/nπ* conical intersection seam, in agreement with a short ππ* lifetime observed in our earlier study^22^. Our intuitive interpretation is supported by our CC investigations of the ππ* and nπ*-state potential energy surfaces. Figure 1b shows a reduced potential energy sketch from our simulations along the two nuclear coordinates, which are expected to be most relevant for the molecular dynamics. We could not observe any accessible local minima in the ππ* state and instead found the ππ*/nπ* conical intersection seam not to be isolated by a barrier from the Franck–Condon (FC) point, but directly accessible. This suggests that after photoexcitation, the nuclear wavepacket is driven out of the FC region by a gradient along the C(5)–C(6) bond elongation towards a saddle point. On its way, it encounters the ππ*/nπ* conical intersection seam, which is in agreement with the experimentally observed internal conversion on a sub-100 fs timescale. In the nπ* excited state, a local minimum can be reached from the ππ*/nπ* conical intersection by O(8)–C(4) bond elongation^33^.

**Fig. 4:**
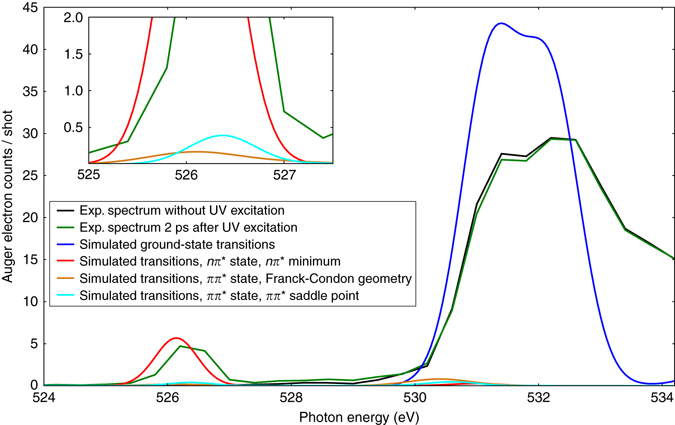
Comparison of experimental and simulated spectra. Simulated near-edge absorption fine structure spectra of the ground state, the ππ* state in the Franck–Condon region and at the saddle point, and the nπ* state at its minimum. For comparison, the experimental ground-state spectrum and the experimental spectrum 2 ps after ultraviolet (UV) excitation are shown. Relative intensities of simulated and experimental ground-state spectra are adjusted to an equal peak area of the π* resonances. Simulated excited-state spectra are scaled with respect to the ground-state spectrum assuming 13% excitation. The *inset* shows a detailed view of the intensity relations at the position of the excited-state feature. Contributions from K-edge ionization were not included in the simulations

We, furthermore, performed CC simulations of NEXAFS spectra of the ground state, and of the excited states at the minimum and saddle point geometries identified in Fig. 1b. Figure 4 shows a comparison of calculated to experimental spectra of the ground state and 2 ps after UV excitation. All three simulated excited-state spectra exhibit their lowest-energy resonance around 526.4 eV. In all cases, the final state is the same O(8)-centered core-excited state (CE). As expected, the oscillator strength in the nπ* state beats the ππ* state by a factor of 40, almost independent of the molecular geometry (Supplementary Tables 1–3). We scaled the simulated excited-state spectra to the estimated ratio of 13% excited molecules. Only the simulated nπ*-state spectrum shows a comparable intensity at the 526.4 eV position.

## Discussion

With the TR-NEXAFS method presented here, we can confirm population of the nπ* state through a directly accessible conical intersection within 60 fs. Comparison of experimentally observed and calculated NEXAFS absorption intensities suggests that internal conversion into the nπ* state is a major channel in the relaxation dynamics of thymine (Supplementary Note 3).

An alternative method to explore the electronic relaxation, however less selectively sensitive to electronic structure changes, is time-resolved photoelectron spectroscopy using extreme ultraviolet pulses^34^. Here one relies on spectrally resolving ionic continua that single out certain valence states^35^, thus exhibiting nonadiabatic transitions by kinetic energy or angular distribution changes^36, 37^. An assignment is, however, only possible by high-level simulations of excited-state ionization cross-sections, whereas we confirm the validity of our intuitive orbital-based assignment of core excitations by quantitative CC simulations.

The nπ* signature in our TR-NEXAFS spectra shows a biexponential decay with time constants of (1.9 ± 0.1) ps and (10.5 ± 0.2) ps. This supports a consecutive relaxation process via a level with nπ* character to a final level of non-nπ* character, to which our method is insensitive. The nπ* level is most probably another minimum in the singlet nπ* state, as intersystem crossing to a triplet nπ* state is forbidden by the El Sayed selection rule. The transition to the final state can be either intersystem crossing to a nearby triplet ππ* state with strong spin orbit coupling^38^ or internal conversion to the ground state^39^, which is supported by the recovery of the ground-state bleach in our data. Recent simulations on ethylene^40^ suggest that 1*s*–*π* absorption lines exhibit geometry-dependent line shifts. We predicted those features to be relatively weak in the case of thymine, especially at the oxygen K-edge. It would be exciting to explore this opportunity in a future experiment with sufficient signal to noise. In a similar way, a recent study on time resolved absorption spectroscopy at the carbon K-edge uses symmetry effects on the X-ray absorption spectrum to probe strong field induced dissociation of SF_6_^41^. This is particularly exciting taking into account that a high harmonic source was used to accomplish this.

In conclusion, we demonstrate with this work the application of time-resolved X-ray absorption spectroscopy to organic molecules containing heteroatoms with lone pairs to selectively investigate ultrafast ππ*/nπ* internal conversion, a crucial mechanistic step in this large class of molecules. We use the dominating absorption strength of the heteroatom 1*s*–*n* resonance to directly monitor this nonadiabatic process. Our results prove that the method already works reliably under the current, still challenging conditions of X-ray free-electron laser experiments with low repetition rates and high temporal and spectral jitter. Application of the method is in principle not confined to the gas phase. A soft X-ray study of molecular desorption using similar technical constraints but different molecular processes has been already performed at surfaces^42^. In addition, recently developed liquid sheets allow for time-resolved solution studies in the water window^43^. Our approach has the potential to become a standard tool for ultrafast investigations at the upcoming second generation of ultrafast X-ray sources, which provide the opportunity to extend the TR-NEXAFS technique towards multidimensional spectroscopy approaches in the soft X-ray regime^44^.

## Methods

### Experimental methods

The experiment was performed at the Linac Coherent Light Source (LCLS) free electron laser (FEL) facility, SLAC National Accelerator Laboratory, at the soft X-ray (SXR) instrument^7, 45^. A schematic representation of the experimental setup is shown in Supplementary Fig. 2. Thymine was purchased from Sigma Aldrich and evaporated by an effusive oven into an ultra high vacuum chamber at a temperature of 160°C leading to a sample density of 10^12^ cm^−3^ in the overlap region of optical and X-ray laser^22, 46^. Molecules were excited by 267 nm pulses with 70 fs duration and a focus diameter of 100 μm full width at half maximum (FWHM). Soft X-ray pulses with 70 fs duration and a focus diameter of 70 μm FWHM were used to probe the sample in the oxygen K-edge spectral region from 520 to 550 eV by simultaneously tuning the FEL and the monochromator of the SXR instrument with an energy resolution of <0.5 eV^47^. The intensity of the essentially background-free transient feature at 526.4 eV was measured for a wide range of UV pump intensities, to make sure the experiment took place in the linear absorption regime well below saturation (Supplementary Fig. 3). Temporal and spatial overlap of UV and SXR pulses was optimized to bleach the Auger spectra of thymine which is induced by photofragmentation at high UV intensities. Oxygen 1*s* Auger spectra were recorded with the 2 m long LCLS-FELCO (LCLS-FEL correlation) magnetic bottle spectrometer^48^. The photon energy-dependent absorption cross-section of the sample is proportional to the integrated Auger electron yield. SXR pulses were delayed with respect to UV pulses between −200 fs and 20 ps. To achieve NEXAFS difference spectra, UV laser pulses were blocked on a shot-by-shot basis. LCLS pulses are strongly fluctuating in intensity and relative arrival time. Therefore, both parameters were recorded on a shot-by-shot basis by an optical X-ray cross-correlator^49^ and a gas detector after the monochromator, respectively. The data set was resorted into ≥50 fs delay bins and several X-ray intensity bins. Difference spectra from different X-ray intensity bins were averaged.

### Theoretical methods

The thymine ground-state geometry (Supplementary Table 4) was optimized with CCSD(T)/aug-cc-pVDZ using CFOUR^50^. Excited-state geometries (Supplementary Tables 5 and 6) were optimized at the EOM-CCSD/aug-cc-pVDZ level employing Q-Chem^51^. No symmetry restrictions were applied for geometry optimizations. Valence excitation energies were obtained with CC3 using the aug-cc-pCVDZ basis on the oxygen atoms and the aug-cc-pVDZ basis on the other atoms (Supplementary Table 7). We employed a newly developed implementation in Dalton^29, 52–54^. Oxygen 1*s* to valence excitation energies and oscillator strengths were computed at the CCSD level of theory with the same basis as for the valence excitations using a newly developed linear response code employing the core-valence separation (CVS) approximation and implemented in Dalton^31, 55–57^ (Supplementary Tables 1–3). This procedure has previously been shown to be highly accurate within the coupled cluster hierarchy of methods. In the CVS approximation, we excluded the pure valence excitations and require the excitation operators to include at least one core orbital—in this way the excitation energies for core-excited states become the lowest eigenvalues of the coupled cluster response matrix. Transition moments are calculated using coupled cluster response theory for transitions between excited states. The core to valence excitation energies was offset-corrected by benchmark calculations of the lowest core to valence excitation energies at the CC3/aug-cc-pCVTZ/aug-cc-pVDZ level (Supplementary Table 8). With this correction, we achieve quantitative agreement with the NEXAFS transition energies within the experimental error bars on a purely ab initio basis. The theoretical core excitation energies are not corrected for relativistic effects and we estimate the effect to increase excitation energies by 0.1–0.3 eV.

Thymine exhibits *C*_s_ symmetry in the ground state. The two lowest-lying excited states have different representations, A″(nπ*) and A′(ππ*), and the ππ*/nπ* conical intersection is symmetry allowed. We note that no complex eigenvalues of the Jacobian matrix were encountered in the vicinity of the conical intersection seam^58^. In contrast to earlier studies^24–26^, we could not identify a minimum in the ππ* state. Instead, we found a saddle point geometry with *C*_s_ symmetry, which is directly accessible from the Franck–Condon region. The energy lowering degrees of freedom of the saddle point are out of plane bending, as confirmed by frequency calculations, which were performed for all observed stationary points^59, 60^ (Supplementary Tables 9–11). The nπ* minimum geometry is distorted from *C*_s_ symmetry with O(8) out of the plane. We encountered the ππ*/nπ* conical intersection seam in between the Franck–Condon point and the ππ* saddle point in close proximity to the latter (energy difference < 0.03 eV). On the basis of calculated core excitation energies and oscillator strengths, NEXAFS spectra were simulated by convoluting theoretical stick spectra with Gaussians to account for peak broadening and the experimental energy resolution.

### Data availability

The data sets generated during and/or analyzed during the current study are available from the corresponding authors on reasonable request.

## Electronic supplementary material

Supplementary Information

Peer Review File
