# Supplementary material for: Probing ultrafast ππ*/nπ* internal conversion in organic chromophores via K-edge resonant absorption
Source: Nat Commun. 2017 Jun 22;8:29. doi: 10.1038/s41467-017-00069-7 (PMC5481431; doi:10.1038/s41467-017-00069-7)
Supplement: Supplementary file 1 — Supplementary Information [file 41467_2017_69_MOESM1_ESM.pdf]

File name: Supplementary Information
Description: Supplementary Figures, Supplementary Tables and Supplementary Notes

File name: Peer Review File
Description:

Supplementary Note 1. Localization of excitations and the single electron picture

For our intuitive prediction of the expected intensities of excited state NEXAFS features we rely on the Hartree-Fock (HF) molecular orbital (MO) based one-electron picture. In this framework, core-excited as well as valence-excited states are described as a single electron excitation from an occupied to an unoccupied HF-MO. Despite its success in the case of relative intensities for core-excitations from the ππ*and nπ* states, it fails in predicting relative intensities for core-excitations from the ground state. The ground state core-excitation leads to the π* MO in the one-electron picture, which is highly delocalized and exhibits only weak density at the oxygens. Nevertheless, both coupled cluster simulations and experimental results predict the transition intensity to be comparable to the nπ* state core-excitation, which involves the strongly localized n MO.

The reason for the failure of the MO picture lies in the HF formalism, which only optimizes occupied MOs. The employed coupled cluster methods calculate valence and core excited states by optimizing a series expansion of electron excitations into the virtual MOs inherently leaving the single electron MO picture. The most appropriate, but less intuitive way to inspect these excitations is therefore to look at electron density changes instead of orbitals (see Supplementary Fig. 1). The density changes, however, qualitatively agree with the MO picture in cases of transitions between MOs which are occupied in the HF reference wavefunction.

Supplementary Note 2. Rate equation model and picosecond dynamics

To analyze the transient 526.4 eV feature in the TR-NEXAFS spectra, a rate equation model was employed, which assumes the following chain of subsequent excited state single exponential population transfers with time constants τ_1 to τ_3:

ππ* state → nπ* state → (nπ*)' state → final state.

Since the 526.4 eV signature decays in a bi-exponential fashion, the step labeled as (nπ*)' had to be included. The character of that step cannot be completely determined by the present results. Its transition moment is only 42 % of the nπ* state according to our fit of the experimental data. It is nevertheless very likely also of nπ* character, since the calculated oscillator strength for core excitation from the ππ* state is only 2.5 % of the oscillator strength from the nπ* state and, thus, one order of magnitude lower. It is most probably another lower lying minimum in the singlet nπ* state, since intersystem crossing to a triplet nπ* state is forbidden by the El Sayed selection rule. Our method is insensitive to the final state, which therefore must have non-nπ* character. It is either the ground state or a ππ* triplet state.

Since the bleach signature only decays slowly within the investigated delay-time window, it is ideal to extract the exact time zero and the instrument response function (90 fs) using an error function fit. To decrease the noise level, a region of interest with the strongest UV-induced modulations was identified in the resonant Auger spectra from the ground state π* resonances. The plotted intensities in Fig. 3 in the main paper refer to the integrated UV-induced changes in this region of interest. For comparison, Fig. 3 in the main paper also shows the analogue signal for the 526.4 eV, which was fitted (see Fig. 2 in the main paper) with a weighted sum of the

time-dependent populations of the nπ* and (nπ*)’ steps, convoluted with the instrument response function (g(t)).

$$I(\Delta t) = g(t) \otimes heaviside(\Delta t) \cdot \left(I_1 \frac{\tau_2}{\tau_2 - \tau_1} \left(e^{-\frac{\Delta t}{\tau_2}} - e^{-\frac{\Delta t}{\tau_1}} \right) + I_2 \frac{\tau_3}{\tau_2 - \tau_1} \left(\frac{\tau_2}{\tau_3 - \tau_2} \left(e^{-\frac{\Delta t}{\tau_3}} - e^{-\frac{\Delta t}{\tau_2}} \right) - \frac{\tau_1}{\tau_3 - \tau_1} \left(e^{-\frac{\Delta t}{\tau_3}} - e^{-\frac{\Delta t}{\tau_1}} \right) \right) \right)$$

The delay between the onsets of the bleach and the 526.4 eV feature is clearly visible in Fig. 3 in the main paper. The time constants τ_2 and τ_3 are (1.9 ± 0.1) ps and (10.5 ± 0.2) ps.

Supplementary Note 3. Excited state population analysis

Calculated and experimental intensities of the nπ* feature and the ground state π* resonance have to be compared, to estimate, which fraction of the excited state population is observable in the nπ* feature. For this, we chose the same NEXAFS spectra of the ground state and 2 ps after UV excitation as in Fig. 2a in the main paper. The ratio of integrated peak areas between the nπ* (I_2) and ground state resonance features (I_1) is 0.053. These can now be compared to the ratio of calculated transition moments for the nπ* feature (σ_2) and the ground state resonance (σ_1) of 0.65. Assuming 100 % population transfer from the ππ* state, the rate equation model predicts the nπ* level to contain 36 % and the (nπ*)’ level 57 % of the overall relative excited state population (P_0). According to the rate equation fit, the transition moment of (nπ*)’ level σ_2' is 42 % of the nπ* transition moment σ_2. This leads to the relation for I_2

$$I_2 = f \cdot P_0 \cdot \sigma_2 \cdot (0.36 + 0.57 \cdot 0.42)$$

where f is a conversion factor between experimental intensities and calculated transition moments. The factor f can be evaluated by $f = \frac{I_1}{\sigma_1}$. Inserting this in the equation for I_2 gives a value of 13 % for the overall excited state population P_0 relative to the ground state. This moderate excitation ratio fits well together with our expectations of the excitation ratio based on our scan of the UV intensity dependence of the nπ* feature intensity. Comparison with the intensity dependence furthermore supports the initial assumption that the relaxation to the nπ* state observed in the present experiment is a major channel for the ππ* population.

Supplementary Note 4. Intensity scan

To confirm that UV excitation takes place in the linear regime, we investigated the dependence of the nπ* feature intensity on the UV intensity (see Supplementary Fig. 3). At low UV intensities, the nπ* feature intensity has a linear response. At high intensities, saturation is observable. The most relevant processes contributing to the excited state population are single photon excitation from the ground state and further single photon excitation from the excited state i.e. sequential or resonance-enhanced two photon excitation. The dependence of the relative population P_0 in the excited state on the photon flux F is therefore

$$P_0 = \frac{\sigma_1}{\sigma_1 - \sigma_2}\left[e^{-\sigma_2 \cdot F} - e^{-\sigma_1 \cdot F}\right]$$

where σ_1 and σ_2 are the ground state and excited state absorption cross-sections. The value of σ_1 is approximately 30 MBarn, the value of σ_2 is unknown. Since absorption of two photons brings the molecule very close to the ionization threshold where the density of states is

particularly high, the value of σ_2 can be expected to be higher than σ_1. The absolute upper limit for relative excited state population can be estimated by neglecting sequential two photon excitations i.e. by setting σ_2 to zero. In this case, the saturation value refers to 100 % population in the excited state. The UV intensity for the pump-probe experiments leads to 24 % of this saturation value. Assuming any value higher than 0 for σ_2 reduces the population in the excited state at saturation. Assuming 13 % excitation at the intensity of the pump probe experiments based on the comparison between experimental and calculated intensities yields a value for σ_2, which is 1.5 times σ_1 and thus perfectly reasonable.

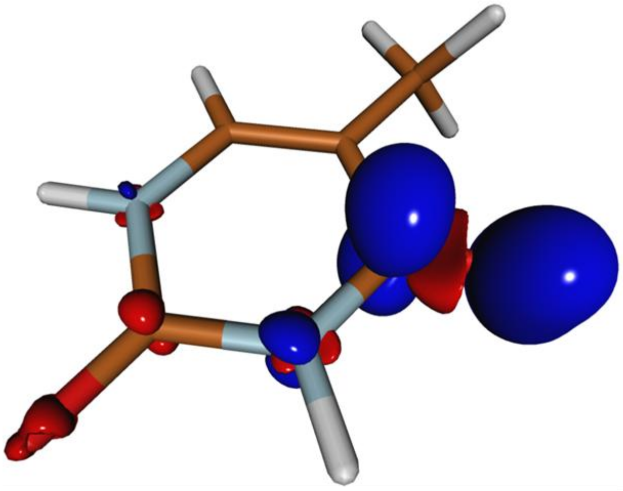

Ground state→CE

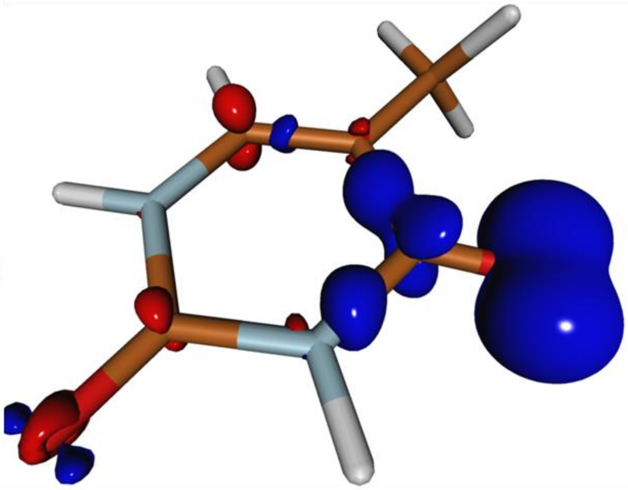

nπ* → CE

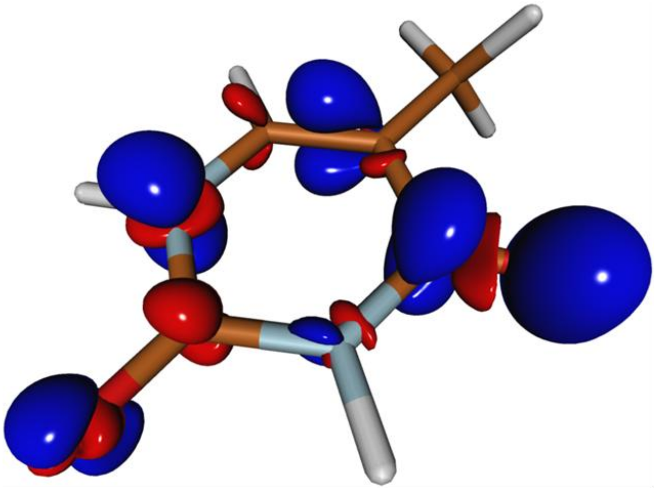

ππ* → CE

Supplementary Figure 1. Visualization of electron density changes for core excitations from the ground state, the nπ* state and the ππ* state to the lowest O(8) 1s core-excited state. In the case of core excitation from the nπ* state and the ππ* state, the electron density changes agree with the predictions from the Hartree-Fock (HF) molecular orbital (MO) based single electron picture. The density change for the nπ* state core excitation is strongly localized at O(8), since two MOs with strong localization at this oxygen are involved. The density change for the ππ* state core excitation is delocalized, since it involves delocalized π MO apart from the localized O(8) 1s MO. The electron density change for the core excitation from the ground state disagrees with the predictions from the HF MO picture. The latter involves the localized O(8) 1s MO and a delocalized π* MO. The electron density change, however, is strongly localized due to linear combination of single electron transitions to several π* MOs.

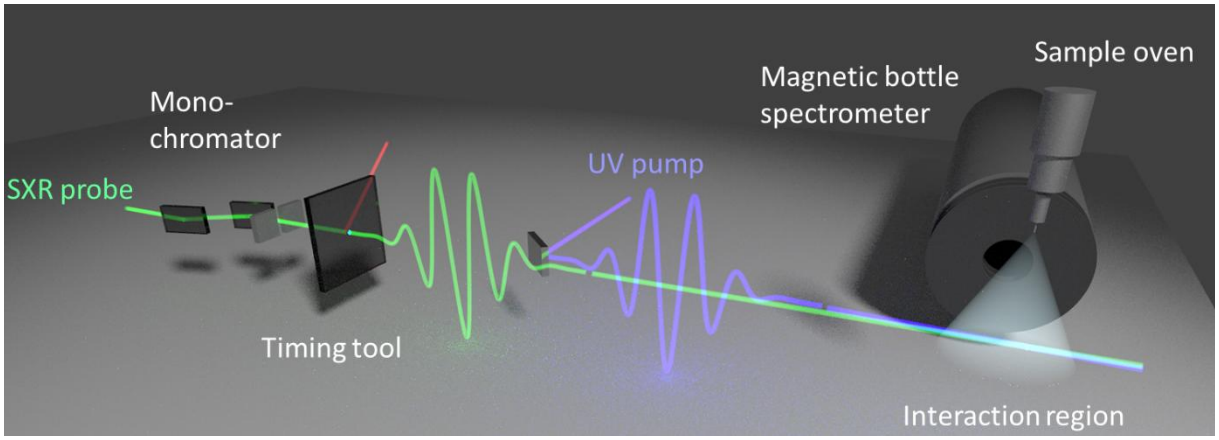

Supplementary Figure 2. Schematic representation of the experimental setup. Broad-bandwidth ultrashort soft x-ray (SXR) pulses (green) are monochromatized and focused into the interaction region of the experimental chamber. There, they are quasi-collinearly overlapped with ultrafast UV pulses with a wavelength of 267 nm (violet). The sample thymine is evaporated into the interaction region by an in-vacuum oven. The relative timing between UV and soft x-ray pulses is measured on a shot-by-shot basis using the SXR timing tool to compensate for the 200 fs timing jitter. Auger electron spectra from core-excited thymine molecules are detected in a magnetic bottle photoelectron spectrometer. NEXAFS spectra can be generated by measuring the photon energy dependent integrated Auger electron yield.

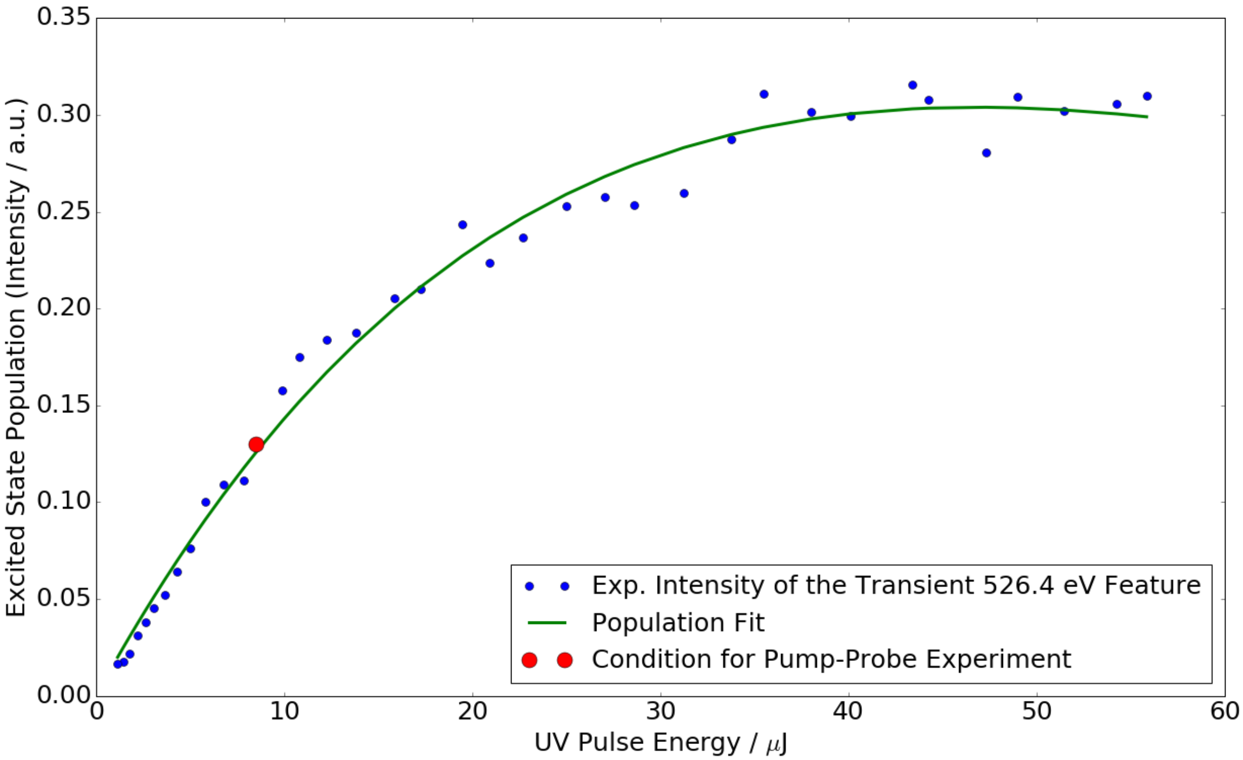

Supplementary Figure 3. Intensity of the transient 526.4 eV feature for different UV intensities together with a rate equation fit. The ordinate is rescaled to match the estimated 13 % excited state population for the UV intensity used in the pump–probe experiment.

Supplementary Table 1. Calculated NEXAFS resonance energies and cross-sections for different states at the Franck-Condon geometry (CCSD/aug-cc-pCVDZ/aug-cc-pVDZ)

O(8) transitions / eV	Oscillator strength	O(7) transitions / eV	Oscillator strength
Ground state			
535.46	3.41E-02	536.45	3.17E-02
538.95	3.60E-04	538.75	1.34E-04
539.86	1.51E-03	539.55	2.39E-03
539.95	2.30E-03	539.70	3.69E-04
539.97	8.26E-04	540.10	1.53E-03
540.27	8.53E-05	540.21	1.01E-03
540.54	1.74E-03	540.42	2.99E-03
540.79	1.11E-04	540.93	5.19E-04
541.05	1.01E-04	541.14	1.00E-03
541.14	6.98E-05	541.44	7.24E-04
nπ* state			
530.56	3.68E-02	531.55	1.10E-06
534.05	8.90E-07	533.85	1.00E-08
534.96	1.20E-06	534.65	1.30E-07
535.05	1.18E-03	534.80	1.20E-03
535.07	1.80E-07	535.20	4.75E-05
535.37	6.15E-04	535.31	2.20E-07
535.64	7.00E-08	535.52	1.00E-08
535.89	1.00E-08	536.03	2.00E-08
536.15	1.40E-07	536.24	1.00E-08
536.24	1.00E-08	536.54	1.27E-05
ππ* state			
530.33	1.74E-03	531.32	3.04E-04
533.82	1.09E-04	533.62	7.22E-05
534.73	3.80E-05	534.42	2.32E-05
534.82	1.17E-03	534.57	6.46E-03
534.84	5.29E-05	534.97	1.61E-04
535.14	9.83E-04	535.08	1.31E-06
535.41	7.09E-06	535.29	4.00E-07
535.66	2.90E-07	535.80	2.10E-07
535.92	1.00E-08	536.01	2.12E-06
536.01	2.33E-06	536.31	4.43E-05

Supplementary Table 2. Calculated NEXAFS resonance energies and cross-sections for different states at the nπ* minimum geometry (CCSD/aug-cc-pCVDZ/aug-cc-pVDZ)

O(8) transitions / eV	Oscillator strength	O(7) transitions / eV	Oscillator strength
Ground state			
534.49	3.24E-02	536.39	3.16E-02
538.53	4.61E-04	538.71	2.32E-04
539.31	2.07E-03	539.05	5.39E-04
539.38	4.47E-03	539.55	2.18E-03
539.45	1.83E-03	540.06	1.45E-03
nπ* state			
530.92	4.32E-02	532.82	1.64E-05
534.96	8.61E-05	535.14	1.67E-05
535.74	5.95E-04	535.48	8.13E-04
535.81	1.06E-05	535.98	6.80E-07
535.88	6.81E-04	536.49	3.25E-06
ππ* state			
529.95	7.73E-03	531.85	3.37E-04
533.99	1.92E-04	534.17	1.36E-04
534.77	1.37E-03	534.51	3.47E-03
534.84	3.96E-04	535.01	1.27E-05
534.91	1.71E-03	535.52	7.42E-06

Supplementary Table 3. Calculated NEXAFS resonance energies and cross-sections for different states at the ππ* saddle point geometry (CCSD/aug-cc-pCVDZ/aug-cc-pVDZ)

O(8) transitions / eV	Oscillator strength	O(7) transitions / eV	Oscillator strength
Ground state			
535.28	3.25E-02	536.42	3.11E-02
538.82	2.55E-04	538.72	7.51E-05
539.64	3.15E-03	539.21	5.46E-04
539.67	1.32E-03	539.51	2.62E-03
539.76	1.48E-03	540.07	1.69E-03
nπ* state			
530.88	3.87E-02	532.02	1.12E-04
534.42	0.00E+00	534.32	1.00E-07
535.24	2.81E-03	534.81	1.50E-03
535.27	5.50E-07	535.11	2.00E-8
535.36	5.10E-07	535.67	1.84E-07
ππ* state			
530.99	2.96E-03	532.13	4.36E-04
534.53	6.56E-05	534.43	6.16E-05
535.35	2.58E-03	534.92	4.85E-03
535.38	4.25E-05	535.22	1.37E-05
535.47	7.67E-05	535.78	7.08E-06

Supplementary Table 4. Ground state minimum geometry (CCSD(T)/aug-cc-pVDZ)

Element	X / Å	Y / Å	Z / Å
C	-1.6332	0.0426	-0.0001
C	0.7570	0.8384	-0.0001
C	1.1979	-0.5713	0.0000
C	0.2400	-1.5432	0.0000
C	2.6817	-0.8510	0.0002
N	-0.6415	1.0215	-0.0007
N	-1.1219	-1.2509	-0.0002
O	-2.8353	0.2919	0.0005
O	1.5118	1.8105	0.0003
H	-0.9734	1.9835	-0.0002
H	0.4869	-2.6087	0.0000
H	-1.8153	-1.9901	0.0005
H	3.1589	-0.4028	-0.8884
H	3.1587	-0.4025	0.8887
H	2.8721	-1.9376	0.0005

Supplementary Table 5. nπ* state minimum geometry (EOM-CCSD/aug-cc-pVDZ)

Element	X / Å	Y / Å	Z / Å
C	1.6756	0.0108	0.0230
C	-0.6393	0.8270	-0.0428
C	-1.1709	-0.4819	-0.0168
C	-0.2637	-1.5185	0.0106
C	-2.6680	-0.6951	-0.0181
N	0.7453	1.0515	0.1200
N	1.1227	-1.2492	0.0249
O	2.8776	0.2283	-0.0350
O	-1.3720	1.9491	0.1211
H	1.1236	1.9641	-0.1066
H	-0.5521	-2.5678	-0.0154
H	1.7941	-2.0025	-0.0250
H	-3.1289	-0.2542	0.8817
H	-3.1341	-0.2281	-0.9015
H	-2.8981	-1.7710	-0.0329

Supplementary Table 6. ππ* state saddle point geometry (EOM-CCSD/aug-cc-pVDZ)

Element	X / Å	Y / Å	Z / Å
N	0.6451	-1.0681	-0.0013
H	0.9407	-2.0373	-0.0009
C	1.6033	-0.1081	0.0001
O	2.8197	-0.2668	0.0006
N	1.1090	1.2319	0.0005
H	1.8635	1.9158	0.0010
C	-0.1892	1.6224	-0.0010
H	-0.4105	2.6884	-0.0014
C	-0.7824	-0.8153	-0.0001
O	-1.5291	-1.8220	0.0005
C	-1.1881	0.5501	-0.0002
C	-2.6474	0.8865	0.0006
H	-2.8068	1.9771	-0.0001
H	-3.1465	0.4513	0.8852
H	-3.1475	0.4502	-0.8830

Supplementary Table 7. Calculated four lowest valence excitation energies at different geometries (CC3/aug-cc-pCVDZ/aug-cc-pVDZ). The electronic character of the excitation is included in brackets.

	Franck-Condon geometry	nπ* minimum geometry	ππ* saddle point geometry
S_1 /eV	4.90 (nπ*)	3.57 (nπ*)	4.29 (ππ*)
S_2 / eV	5.13 (ππ*)	4.54 (ππ*)	4.40 (nπ*)
S_3 / eV	5.65 (πn*)	5.08 (ππ*)	5.26 (πn*)
S_4 / eV	6.17 (ππ*)	5.69 (πn*)	5.60 (ππ*)

Supplementary Table 8. Calculated ground state NEXAFS resonance energies at different geometries (CC3/aug-cc-pCVTZ/aug-cc-pVDZ)

	Franck-Condon geometry	nπ* minimum geometry	ππ* saddle point geometry
Lowest O(8) transition / eV	531.19	529.74	530.62
Lowest O(7) transition / eV	532.23	531.82	531.84

Supplementary Table 9. Vibrational frequencies and IR intensities of the ground state minimum (CCSD(T)/aug-cc-pVDZ)

Frequency / cm^{-1}	IR intensity / km mol^{-1}
100	0.00
134	0.72
143	0.01
269	2.41
279	0.12
376	22.94
381	21.25
451	18.44
525	52.61
536	6.68
594	1.37
665	88.91
730	4.42
735	25.94
754	1.88
797	3.42
875	19.64
960	11.63
1011	1.94
1052	0.12
1154	9.39
1199	154.13
1249	3.83
1373	12.10
1386	0.06
1403	6.08
1424	66.38
1453	6.06
1477	1.66
1499	111.43
1699	0.03
1739	555.62
1778	817.99
3034	23.89
3109	10.51
3123	14.47

3212	4.81
3594	60.42
3643	96.73

Supplementary Table 10. Vibrational frequencies and IR intensities of the nπ* state minimum (EOM-CCSD/aug-cc-pVDZ)

Frequency / cm^{-1}	IR intensity / km mol^{-1}
82	2.64
100	1.89
135	0.51
181	19.25
247	4.70
262	2.22
337	4.95
406	30.45
460	21.14
468	91.00
510	15.10
521	47.83
576	6.25
637	47.10
741	33.82
753	3.86
794	7.54
942	26.29
1012	1.58
1060	0.32
1146	62.73
1211	44.59
1243	13.73
1283	5.37
1390	84.14
1419	0.70
1432	13.49
1476	22.63
1476	6.40
1492	0.86
1516	27.52
1627	45.54
1800	742.28
3048	24.42
3115	13.00
3144	13.12

3265	1.64
3637	70.62
3680	76.92

Supplementary Table 11. Vibrational frequencies and IR intensities of the ππ* state saddle point (EOM-CCSD/aug-cc-pVDZ)

Frequency / cm^{-1}	IR intensity / km mol^{-1}
292i	31.79
126i	0.25
94i	13.22
129	0.54
153	0.30
271	7.96
300	5.65
372	18.71
440	35.66
441	76.76
496	11.09
578	0.36
610	0.16
662	4.14
726	12.06
754	65.88
768	9.75
925	17.76
968	4.02
996	0.39
1150	59.95
1185	30.92
1254	12.70
1319	32.57
1351	11.99
1387	90.04
1412	41.87
1443	7.86
1466	22.10
1480	36.99
1524	1.79
1620	263.15
1762	320.77
3022	14.56
3080	11.34
3121	14.83

3275	5.94
3588	68.66
3650	99.19
