# Supplementary material for: Probing ultrafast ππ*/nπ* internal conversion in organic chromophores via K-edge resonant absorption
Source: Nat Commun. 2017 Jun 22;8:29. doi: 10.1038/s41467-017-00069-7 (PMC5481431; doi:10.1038/s41467-017-00069-7)
Supplement: Supplementary file 2 — Peer Review File [file 41467_2017_69_MOESM2_ESM.pdf]

Editorial Note: this manuscript has been previously reviewed at another journal that is not operating a transparent peer review scheme. This document only contains reviewer comments and rebuttal letters for versions considered at Nature Communications.

REVIEWERS' COMMENTS:

Reviewer #1 (Remarks to the Author):

This paper from Guehr and co-workers has been modified from their initial submission to Nature in an entirely appropriate manner. I am happy to recommend for publication as is.

Reviewer #2 (Remarks to the Author):

The authors have addressed my comments and I have no further issues I would like to see addressed. I believe in addition that their response to the comments from Referee #1 have strengthened the paper. I recommend publication in Nature Communications.
